# Aerosolized Niosome Formulation Containing Gemcitabine and Cisplatin for Lung Cancer Treatment: Optimization, Characterization and In Vitro Evaluation

**DOI:** 10.3390/pharmaceutics13010059

**Published:** 2021-01-05

**Authors:** Norfatin Izzatie Mohamad Saimi, Norazlinaliza Salim, Noraini Ahmad, Emilia Abdulmalek, Mohd Basyaruddin Abdul Rahman

**Affiliations:** 1 Integrated Chemical BioPhysics Research, Faculty of Science, Universiti Putra Malaysia, Serdang 43400 UPM, Selangor, Malaysia; norfatinizzatiesaimi@gmail.com (N.I.M.S.); emilia@upm.edu.my (E.A.); basya@upm.edu.my (M.B.A.R.); 2 Department of Chemistry, Faculty of Science, Universiti Putra Malaysia, Serdang 43400 UPM, Selangor, Malaysia; 3 Department of Chemistry, Faculty of Science, University of Malaya, Kuala Lumpur 50603, Malaysia; ainie@um.edu.my; 4 UPM-MAKNA Cancer Laboratory, Institute of Bioscience, Universiti Putra Malaysia, Serdang 43400 UPM, Selangor, Malaysia

**Keywords:** niosome, gemcitabine, cisplatin, heating method, lung cancer, aerosol output

## Abstract

Gemcitabine (Gem) and cisplatin (Cis) are currently being used for lung cancer treatment, but they are highly toxic in high dosages. This research aimed to develop a niosome formulation containing a low-dosage Gem and Cis (NGC), as an alternative formulation for lung cancer treatment. NGC was prepared using a very simple heating method and was further optimized by D-optimal mixture design. The optimum NGC formulation with particle size, polydispersity index (PDI), and zeta potential of 166.45 nm, 0.16, and −15.28 mV, respectively, was obtained and remained stable at 27 °C with no phase separation for up to 90 days. The aerosol output was 96.22%, which indicates its suitability as aerosolized formulation. An in vitro drug release study using the dialysis bag diffusion technique showed controlled release for both drugs up to 24 h penetration. A cytotoxicity study against normal lung (MRC5) and lung cancer (A549) cell lines was investigated. The results showed that the optimized NGC had reduced cytotoxicity effects against both MRC5 and A549 when compared with the control (Gem + Cis alone) from very toxic (IC_50_ < 1.56 µg/mL) to weakly toxic (IC_50_ 280.00 µg/mL) and moderately toxic (IC_50_ = 46.00 µg/mL), respectively, after 72 h of treatment. These findings revealed that the optimized NGC has excellent potential and is a promising prospect in aerosolized delivery systems to treat lung cancer that warrants further investigation.

## 1. Introduction

Over the past fifty years, major public health concern for cancer diseases has been rising, especially for lung cancer, which is the most common cancer diagnosed in the world, contributing to 37% of the leading causes of cancer-related death worldwide from total deaths in 2018 [1]. There are two main categories of lung cancer—non-small cell lung carcinoma (NSCLC), which makes up 85% of cases, while small-cell lung carcinoma (SCLC) amounts to 15% of cases among all lung cancer cases [2]. A significant factor that leads to lung cancer is cigarette smoking. However, increasing lung cancer cases among non-smokers are quite alarming nowadays [3].

Different stages of lung cancer with different treatments are the primary reason for the low survival rate of cancer patients. Likewise, in other cancer cases, detection and confirmation usually can only be made at stage four (IV) [4]. The current treatments used for lung cancer are surgery, chemotherapy, and radiation therapy depending on the stage and overall performance of cancer cells. For the advanced stage of lung cancer, chemotherapy is the first-line treatment, where most of the treatments are available as intravenous (IV) formulations into systematic circulation [5]. Cancer chemotherapeutic drugs are usually used to destroy cancer cells by preventing the cancer cell from growing, dividing, and producing more cells. Conventionally, a patient who undergoes chemotherapy will be given a single or multi-drug at a time—for example, either Cis or Gem alone or a combination of both drugs. It was found that the combination of drugs improved the survival rate among cancer patients compared to single drug usage [6]. This has shown that the optimum dosage of the combination of drugs can be efficient in increasing the survival rate of the lung cancer patient as well as help minimize side effects such as vomiting, sore throat, chest congestion, and nausea [7].

Generally, Gem and Cis are administered at a higher dosage of 1.250 and 0.075 g/m^2^ per cycle, respectively, which will lead to higher toxicity [8]. Cancer chemotherapeutic drugs that are administered orally are always limited due to the first-pass metabolism, where the drug concentration is usually reduced as it passes through the gastrointestinal tract, before the drug can access the systematic circulation [9]. Multi-drug resistance (MDR) in lung cancer stem-like cells (CSC) becomes a limitation to the combination of cancer therapy even though the dual drug has the potential to improve cancer treatment by reducing the drug dosage which leads to less side effects [10]. The suitability of the liposomal Gem-HCl formulation in dry powder form, developed by Gandhi et al. [11], was confirmed for lung cancer through in vitro aerosol delivery with deposition of formulation around the trachea and upper portion of the lung. As studied by Levet et al. [12], a dry powder form of Cis also showed deposition of formulation in the deeper lung with controlled-release of Cis at high doses. Thus, a more efficient carrier for this combination of drugs should be developed to improve the delivery of both drugs to the lung (targeted site).

The demand for nanocarriers for drug administration in medical applications, especially in chemotherapy treatment, has increased as drug development is advancing. Nanocarriers are believed to have great potential in the delivery of single or multiple drugs (at low-dosage drug) to tissue tumors by overcoming biological barriers and can reach the tiniest area in the body due to their small particle size. Furthermore, most of the nanocarrier systems increase the effectiveness of chemotherapy and minimize side effects by enhancing the deposition of the drug at the tumor based on their stability and permeability [13]. Nanocarriers provide a larger surface area, have the potential to increase solubility, improve the controlled release of the drug, and enhance the bioavailability in the delivery of chemotherapy drugs to the targeted tumor area. There are a few nanocarriers currently being used as targeting drug delivery systems, such as micelles, dendrimers, nanoparticles, liposomes, and niosomes [14].

Among these nanocarriers, liposomes and niosomes have great potential in multiple drug delivery. Niosomes are formed of bilayer spherical vesicles, which consist of nonionic surfactants and cholesterol in an aqueous medium [15], while liposomes are bilayer vesicles made by phospholipids enclosing an aqueous medium [16]. Both have efficiency in encapsulating hydrophilic and hydrophobic drugs at the aqueous layer and the lipid bilayer, respectively. However, liposomes are less suitable because of their instability due to oxidation or hydrolysis of phospholipids, they need special storage conditions in a dark area sealed with nitrogen, and the materials used to produce liposomes are expensive [17]. Nonionic surfactants are commonly used in the preparation of niosomes due to their high degree of compatibility with other ingredients. As membrane additives to the bilayer composition of niosomes, cholesterol enhances the stability and reduces the leakage of vesicles, which will increase the entrapment efficiency of the drug [18]. Niosomes are chemically stable, have high compatibility with biological systems, and low toxicity because of their nonionic nature [19].

Therefore, it is not surprising that niosomes (as nanocarrier) are currently being used to deliver drugs such as (i) 5-Fluorouracil, increasing drug penetration in skin cancer treatment; (ii) tamoxifen citrate, providing higher cytotoxicity against breast cancer cells; and (iii) curcumin, giving more significant apoptotic effect towards ovarian cancer cells [20]. However, there is no study which has been conducted on dual drug usage (Cis and Gem) to treat lung cancer via aerosolization. Thus, this research aims to prepare and optimize a niosome formulation intended for aerosol delivery containing Gem and Cis using Mixture Experimental Design (MED). The physicochemical characterization, aerosol output, and stability of the optimized niosome formulation were investigated. The in vitro drug release and cytotoxicity study towards lung cancer (A549) cell lines were also evaluated.

## 2. Materials and Methods

### 2.1. Materials

Gem and Cis were purchased from AK Scientific (Ahern Avenue, Union City, CA, USA). Sorbitan monostearate (Span 60) and polyoxyethylene sorbitan tristearate (Tween 65) were purchased from Fluka (Loughborough, UK). Sodium dodecyl sulfate (SDS), sodium chloride (NaCl), and acetone were purchased from Merck (Darmstadt, Germany). Cholesterol, phosphate buffered saline (PBS, pH 7.4), and simulated lung fluid (SLF, pH 7.4 and 6.7) were supplied by R & M Chemical (Selangor, Malaysia). Glycerol was purchased from Fisher Scientific (Loughborough, UK). Deionized water was from a Milli-Q filtration system, EMD Millipore (Billerica, MA, USA).

### 2.2. Drug Solubility Study

A combination of drug Gem and Cis (0.10% *w*/*w*) with ratio 1:1 was added into 5.00 g of NaCl solution (0.90% *w*/*v* in deionized water). The mixture was stirred in a water bath at 1000 rpm, 27 °C for 15 min using the water bath technique on a magnetic stirrer (MS-H280-Pro, Scilogex LC, Rocky Hill, CT, USA) until all the Gem and Cis were dissolved and a clear NaCl solution was observed. Another 0.10% *w*/*w* of Gem and Cis was added into the previous NaCl solution until a clear solution was formed. The step of Gem and Cis addition (0.10% *w*/*w*) was repeated until a non-clear and precipitation of the drug in NaCl solution was observed. The total percentage of the combination of Gem and Cis that successfully dissolved in the clear NaCl with no precipitation signs after centrifuging using a centrifuge (EBA 200, Hettich, GmbH & Co., KG, Tuttlingen, Germany) at 4000 rpm for 15 min was recorded as the highest amount of gemcitabine and cisplatin that can be dissolved in NaCl solution.

### 2.3. Preparation of Niosome Formulation

NGC was prepared according to the heating method described by Basiri et al. [21] with some modifications. For the lipid phase, a combination of surfactants (Tween 65: Span 60 with a ratio of 1:2) was mixed using a magnetic stirrer at 800 rpm, 120 °C for 5 min in an amber glass Schott bottle. Cholesterol was added into the mixture and further homogenized for 30 min. SDS was then added into the mixture, and then, continuously heated at 120 °C for 30 min. Glycerol solution (3% *w*/*w* in PBS pH 7.4) was added into the mixture dropwise and then, continuously heated at 60 °C for 45 min. The final mixture was sonicated using a bath sonicator (Powersonic 405, Hwashin Technology, Seoul, Korea) at 27 °C for 15 min.

For the aqueous phase, Gem and Cis were mixed in a NaCl solution (0.90% *w*/*v*), which had been sonicated at 27 °C for 15 min. Then, the mixture was stirred for 15 min using a magnetic stirrer at 1000 rpm. The mixture was added into the lipid phase and continuously homogenized by a magnetic stirrer at 1000 rpm, 60 °C for 60 min. Finally, the sample was sonicated using a probe sonicator (Vibra Cells, Sonics, Newtown, CT, USA) for 30 s “on” and 5 s “off” for four cycles.

### 2.4. Optimization of the Niosome Formulation

#### 2.4.1. Experimental Design

The niosome formulation was optimized using the D-optimal mixture design according to the method used by Arbain et al. [22]. The results were statistically analyzed using Design-Expert, Version 7.0 (Stat-Ease Inc., Minneapolis, MN, USA, 2005) and Statistica, Version 12 (Statsoft Inc., Tulsa, OK, USA, 2013). In this preliminary study, the lower and the higher limits of the independent variables were determined by the software. The minimum and maximum proportions of these components are listed in Table 1, while Cis (0.05% *w*/*w*), Gem (0.05% *w*/*w*), NaCl solution (33.33% *w*/*w*), and SDS (0.50% *w*/*w*) were kept constant. The independent variables were utilized to investigate the effect of percentage of surfactant, cholesterol, and glycerol solution on a response variable (particle size) of niosome formulation. The three-dimensional surface graphs were plotted to show the effect of independent variables on the response variable. The optimal compositions for niosome formulation with minimum response variables were chosen.

#### 2.4.2. Statistical Analysis

Analysis of variance (ANOVA) and *R*^2^ (coefficient of determination) were carried out to investigate the significant differences among the independent variables. In order to obtain the final model with a very high correlation, Prob > F value must be significant (<0.05) and the value of *R*^2^ must be higher than 0.9. A non-significant lack of fit and desirable adequate precision greater than 4.00 indicated that the model could be used to navigate the space.

#### 2.4.3. Verification of Models

The optimum composition was selected from the designed model by the D-optimal mixture design that gives a minimum particle size. Several random formulations were prepared according to the range variables to validate the obtained models and compare the actual and predicted values using Equation (1):

(1) $$\mathrm{Residual}\;\mathrm{Standard}\;\mathrm{Error}\;(\%\mathrm{RSE})\;=\;(\frac{\mathrm{Experimental}\;\mathrm{value}\;-\;\mathrm{Predicted}\;\mathrm{value}}{\mathrm{Predicted}\;\mathrm{value}})\;\times \;100\%$$

### 2.5. Physicochemical Characterization

#### 2.5.1. Particle Size, Polydispersity Index (PDI) and Zeta Potential Measurement

Particle size, polydispersity index, and zeta potential were measured using the Dynamic Light Scattering (DLS) technique which scattered at the angle of 173° and temperature of 25 °C. This process was carried out using the Malvern Nano ZS90 (Malvern Instrument, Malvern, UK). Niosome formulation was diluted with PBS (1:100) and injected into the sample cell. The measurement was repeated in triplicate. The calculation of zeta potential was performed based on the measurement of the electrophoretic mobility of dispersed particles in a charged field.

#### 2.5.2. pH Measurement

The pH of the niosome formulation was measured using a Delta 320 pH meter (Mettler-Toledo, Schwerzenbach, Switzerland) at room temperature. The pH meter was calibrated with pH standard buffer solutions before measurements. The average value of pH from the three readings was calculated.

#### 2.5.3. Surface Tension Measurement

The surface tension of niosome formulation was determined using a KSV Sigma 702 tension meter (Biolin Scientific, Espoo, Finland) by a ring method. Before measuring, the platinum ring was cleaned with acetone and washed thoroughly with deionized water, then burnt red using the Bunsen burner. The Du Noüy ring method was applied. Therefore, calibration with the deionized water must be taken at 72 mN/m. The ring was submerged in the sample and then, slowly lifted out of the liquid. The average of the surface tension values was calculated (*n* = 3).

### 2.6. Drug Entrapment Efficiency Measurement

The entrapment efficiency (EE %) can be determined using centrifugation of 1 mL of the formulation at 21,000 rpm for 1 h. The supernatant and the sediment were separated and then, diluted to 25 mL with SLF pH 7.4, filtered using a PTFE syringe filter (0.22 μm pore size, Membrane Solutions, Auburn, WA, USA), and then, measured using a UV–vis spectrophotometer (Beckman, DU 530, Fullerton, CA, USA). The percentage of drug entrapment in niosomes was calculated using Equation (2).

(2) $$\mathrm{Entrapment}\;\mathrm{efficiency}\;(\mathrm{EE}\;\%)\;=\;(\frac{\mathrm{Total}\;\mathrm{drug}\;-\;\mathrm{Drug}\;\mathrm{in}\;\mathrm{the}\;\mathrm{supernatant}}{\mathrm{Total}\;\mathrm{drug}})\;\times \;100\%$$

### 2.7. Morphology

Transmission Electron Microscopy (TEM) was performed to investigate the morphology of niosome by placing a drop of niosome suspension onto carbon-coated copper grids (400-mesh pores). Niosome formulation was diluted with PBS, pH 7.4 (1:100). The sample was then negatively stained with 1% uranyl acetate for 10 min at room temperature. After the excess liquid was drained off with a filter paper, the grid containing niosome samples was observed by a Hitachi H-7100 Transmission Electron Microscope (Tokyo, Japan). 

### 2.8. Aerosol Output

The aerosol output of the optimized niosome was evaluated according to the method used by [23]. A total of 3.0 mL of optimized niosome was loaded in the medication compartment of an OMRON MicroAIR nebulizer (NE-U22V1, Kyoto, Japan) and was commenced to dryness on continuous mode. Aerosol output was calculated before and after nebulization (Equation (3)).

Aerosol output (%) = (W_initial_ − W_final_)/W_initial_ × 100 (3)

where W_initial_ was the weight of liquid present in the nebulizer before nebulization and W_final_ was the weight of liquid remaining after nebulization.

### 2.9. Stability Study

The optimized niosome formulation underwent a centrifugation test at 4000 rpm for 15 min. The sample was divided into three test tubes and kept at different storage temperatures (4, 27, and 45 °C) for 90 days. The physical appearance (no phase separation and no change in color), changes in particle size, and PDI with time were observed. The graphs of the particle size as a function of time at different temperatures were plotted.

### 2.10. In Vitro Drug Release Study

The diffusion of formulations was studied using the dialysis bag diffusion technique. Cellulose membranes were soaked overnight in the release medium. Then, 6.50 mL (M°_Cis_ = 3.75 mg, M°_Gem_ = 3.75 mg) of optimized niosome formulation was placed into the dialysis bag. Both ends of the bag were tied and then carefully immersed in a beaker containing a simulated lung fluid at pH 7.4, which mimicked the normal lung’s pH condition. The elution medium was stirred using a magnetic bar at 100 rpm. One milliliter of the receptor medium was withdrawn at different time intervals (typically at 1, 2, 3, 4, 5, 6, 7, 8, 10, 12, and 24 h) and then, replaced with the same volume of fresh media to maintain the sink conditions. These procedures were repeated with simulated lung fluid pH 6.7, which mimicked the pH condition of lung cancer. All experiments were carried out in triplicate.

These samples were analyzed using a UV–vis spectrophotometer (Beckman, DU 530, Switzerland) at ʎ_max_ 207 nm for Cis and ʎ_max_ 268 nm for Gem to determine the amount of both drugs released. The calibration curves for Cis and Gem in simulated lung fluid at pH 7.4 and in simulated lung fluid at pH 6.7 were plotted separately to determine cumulative percentage of Cis and Gem released. The amount of drug released from NGC was calculated using the following equation:

(4) $$\mathrm{Drug}\;\mathrm{released}\;(\%)\;=\;\frac{M_{d}\;\times \;V\;\times \;100}{{M{}^{\circ}}_{d}}$$

where M_d_ is the concentration of Cis or Gem in the supernatant measured spectrophotometrically at λmax of Cis or Gem, V is the volume of the release media, and M°_d_ is the amount of Cis or Gem loading.

#### Kinetic Release Measurement

The following mathematical models were used to evaluate the kinetics and mechanism of drug release from optimized niosome formulation: zero-order (cumulative amount of drug release versus time, Equation (5)), first-order (log the cumulative amount of drug remaining versus time, Equation (6)), Higuchi (cumulative percentage of drug release versus square root of time, Equation (7)), Hixson–Crowell (cube root cumulative amount of drug remaining versus time, Equation (8)), and Korsmeyer–Peppas (log cumulative percentage of drug release versus log time, Equation (9)). The model that best fit the drug release data was selected based on the correlation coefficient (*R*^2^) value obtained from the plotted graph in various models. The model that gave the highest value was considered as the best fit for release data.

(5) $$M_{t}{\;=\;M}^{0}{+\;k}^{0}t$$

(6) $$\ln\;M_{t}\;=\ln\;M^{0}{\;+k}^{1}t$$

(7) $$M_{t}{\;=\;k}_{H}\sqrt{t}$$

(8) $$M^{0}{}^{\frac{1}{3}}\;-\;{M_{t}}^{\frac{1}{3}}{=\;k}_{w}t$$

(9) $$M_{t}/\underset{\infty }{M}{=\;k}_{m}{\;t}^{n}$$

where $M^{0}$ is the initial amount of drug in dissolution media, M_t_ the amount of drug released in time, $k^{0}$, $k^{1}$, $k_{H}$, $k_{m}$ are the release rate constants, $M_{t}/\underset{\infty }{M}$ the fraction of drug release over time, n is the release exponent, and t is the time.

### 2.11. In Vitro Cytotoxicity Study on Normal Lung (MRC5) and Lung Cancer (A549) Cell Lines

MTT assay (3-[4,5-dimethylthiazol-2-yl]-2,5-diphenyltetrazolium bromide) on normal lung (MRC5) and lung cancer (A549) cell lines were used to determine the cytotoxicity of the optimized NGC. A concentration of 2 × 10^3^ cells/mL was prepared and plated (100 µL/well) onto 96-well plates. The samples were diluted onto each well with identified concentrations 100.000, 50.000, 25.000, 12.500, 6.250, 3.125, and 1.560 μg/mL, and then, were further incubated for 72 h. MTT solution was added to each cell and continued for incubation in the incubator for 3 h. After solubilization of the purple formazan crystals using DMSO was completed, the Optical Density (OD) of the samples was measured using an ELISA reader at a wavelength of 570 nm. Cytotoxicity was recorded as the drug concentration causing 50% growth inhibition of the cells (IC_50_ value) using Equation (10), given below:

(10) $$\mathrm{Cell}\;\mathrm{viability}\;=\frac{\;\mathrm{Absorbance}\;\mathrm{of}\;\mathrm{sample}}{\mathrm{Absorbance}\;\mathrm{of}\;\mathrm{control}}\;\times \;100\%$$

After determination of the percentage of cell viability was completed, graphs of the percentage of cell viability against their respective concentrations were plotted. This experiment was repeated using a blank niosome formulation (without drugs).

#### Statistical Analysis

By using Statistical Analysis System (SAS) software version 4.3 (SPSS Inc., Chicago, IL, USA, 2013), the statistical analysis of variance (ANOVA) of the experiment was determined using two tests, which were a Type I *t*-test (least significant different, LSD) and Duncan’s Multiple Range Test. A *p*-value of less than 0.05 was considered significant. All experiments were carried out in triplicate. The mean values with standard deviation (SD) were represented as an error bar.

## 3. Results and Discussion

### 3.1. Drug Solubility

The study of drug solubility plays an important role in determining the maximum amount of drugs, Gem and Cis, that can dissolve in 0.90% *w*/*v* NaCl solution, which acts as an aqueous medium in NGC formulation. Table 2 shows that the maximum mixture of Gem and Cis could be solubilized in NaCl solution was 0.30% *w*/*w*. Both Gem and Cis are hydrophilic drugs. It would be encapsulated in the aqueous phase of the niosomes. Hydrophilic drugs are usually subjected to instability, low permeability across barriers, fast drug clearances from the body system, short half-life in the circulatory system, and always come with toxic side effects [24]. Thus, the niosome acts as a water-based vehicle which could provide both a hydrophilic and hydrophobic environment and could be the potential nanocarrier to overcome those problems.

### 3.2. Optimization of Niosome Formulation

#### 3.2.1. Experimental Design and Model Fitting

All NGC formulations were prepared and optimized according to the experimental design. The effects of independent variables: surfactants, cholesterol, and glycerol solution on a response particle size were recorded (Table 3). The combination of these independent variables resulted in an observable change in the particle size of niosomes, varying between 160.20 and 255.10 nm. The actual particle size values obtained experimentally agreed with the predicted particle size values from 16 run experiments, which fitted to the special cubic model using Design-Expert with RSE values of less than 5.00%.

The final reduced model had a significant *p*-value (*p* < 0.0001) (Table 4). For this response, the special model presented less SD but higher values of *R*^2^ and Adj-*R*^2^ (~1.000), which estimates the amount of variation in the data accounted for the model.

The final equations for the model describing the particle size can be written as the following equation:

Particle size = −19,783.33 A + 1.202 × 10^5^ B − 3.20 C + 18,114.43 AB + 332.04 AC − 1842.37 BC − 334.52 ABC (11)

The regression equation of the particle size of the niosome clearly explains that the impact of surfactants (A) and glycerol solution (C) is inversely related to the particle size, while cholesterol (B) possesses a directly proportional effect to the particle size of niosome. The interactions of parameter A with B and A with C are unfavorable, while the interactions of parameter B with C and A with B are favorable in increasing and decreasing the particle size of niosomes, respectively.

#### 3.2.2. D-Optimal Analysis

**Figure 1:**
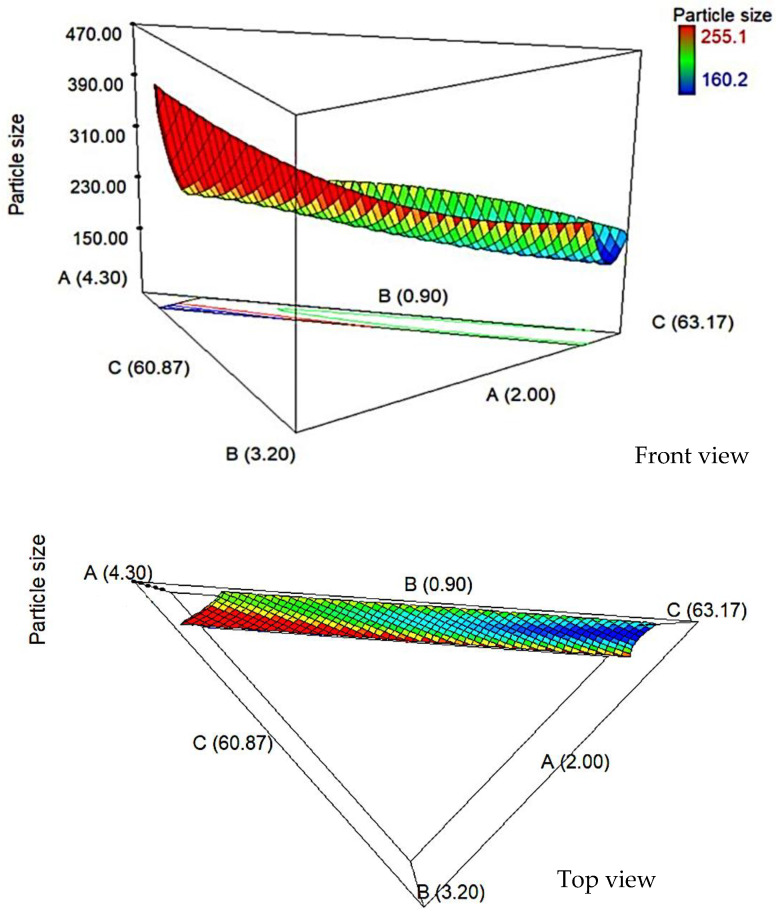
3D surfaces showing the interaction effect of the surfactants (A), cholesterol (B), and glycerol solution (C) against particle size.

The surfactant controls the growth of vesicles and offers adsorption, while preventing agglomeration. Figure 1 shows that as the percentage of surfactant increased, the particle size was found to decrease. As the percentage of surfactant increased from 2.00 to 3.00% *w*/*w*, the particle size was found to decrease from 191.60 to 182.20 nm. This may be due to decreasing of the interfacial tension and reducing of the Laplace pressure and stress by increasing the percentage of surfactant, which leads to a smaller particle size [25]. However, further increases in surfactant up to 4.00% *w*/*w* increased the particle size to 241.70 nm due to an imbalance of lipid composition with the cholesterol, which would affect the physical properties of the vesicle.

Decreasing the cholesterol percentage from 0.90 to 1.20% *w/w* caused the particle size to increase from 191.60 to 255.10 nm. High percentage of cholesterol will increase the hydrophobicity of the bilayer membrane, which produced the disturbance in the vesicular membrane. Hence, it increased the vesicle radius between the bilayer membrane. At a low percentage of cholesterol, the cholesterol will be closed, packing with a surfactant monomer to reduce the vesicle [26].

Altering the percentage of glycerol solution (acts as a hydration medium) in preparation of NGC, which also may affect the particle size of niosome. The 63.02% *w*/*w* of glycerol solution produces a smaller particle size (166.4 nm) when compared with the larger particle size of niosome, 231.9 nm, where the percentage much lower of 60.87% *w*/*w*. A high percentage of glycerol solution, producing a smaller particle size, may be due to high intermolecular bonding between the components and the hydration medium. Yeo et al. [27] also found that niosomes containing Span 60: Cholesterol: Cremophor® ELP with 5 mL hydration volume and 60 min hydration time produced smaller niosome particle sizes and more stable niosomes compared to shorter hydration time of 15 min and lower volume of 2.5 mL. However, longer hydration time contributed to reduce in niosomes particle size despite different hydration volumes. 

#### 3.2.3. Verification of Model

The significance of the fitted model can be proved by performing randomization of a few niosome formulations to confirm that the model fit. Evaluation of the percentage of residual standard error (RSE%) was determined by comparing the actual and predicted particle size of all the randomized formulations. Table 5 shows that there is no significant difference between the actual and predicted values of the particle size of the niosome with RSE values below 2.00%, which indicated that the model fit to the system.

Evaluation of the interaction effect between independent variables (surfactant, cholesterol, and glycerol solution) toward particle sizes as a response was performed analytically in order to obtain a robust prediction for optimized niosome composition. The optimized niosome was selected from the D-optimal mixture design analysis based on set criteria of minimum particle size. Based on the model predicted by the Design-Expert software, the optimum composition of niosome formulation was obtained, as stated in Table 6. Then, the suggested composition by the software was used to prepare NGC and further characterized to achieve the target goal application of the aerosolized formulation to the lung.

### 3.3. Physicochemical Characterization

#### 3.3.1. Particle Size, Polydispersity Index (PDI), and Zeta Potential

**Figure 2:**
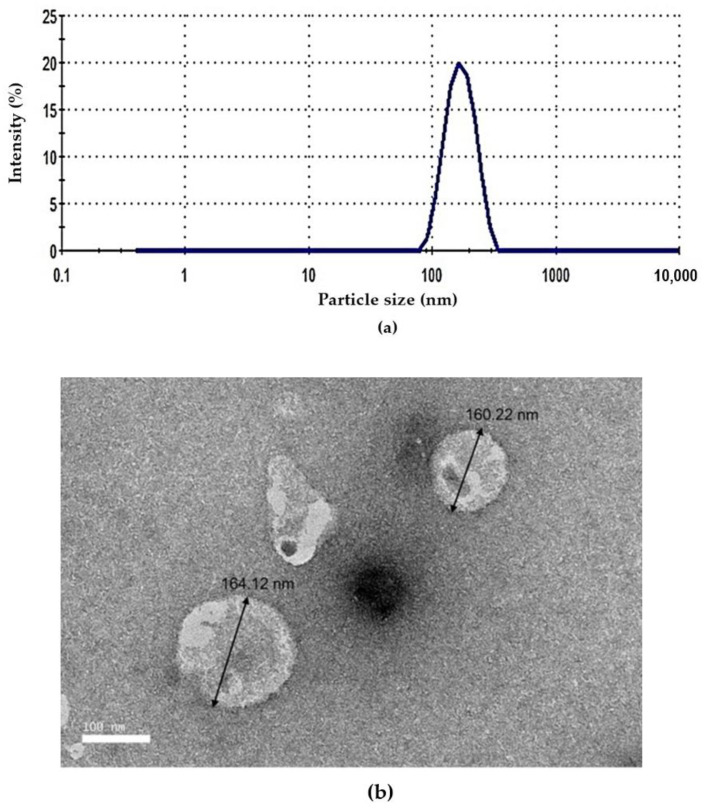
(**a**) The representative of the particle size distribution and (**b**) TEM images of the optimized NGC formulation at 25,000× magnification.

The particle size of optimized NGC was 166.45 nm; the niosome formulation meets the requirement for the aerosolized application, as a particle size that was suitable to be aerosolized was in the range of 50–500 nm [28]. PDI value describes the uniformity of particle distribution in the niosome system. The obtained PDI was 0.16 ± 0.02, which is considered as a monodispersed sample. A small PDI value indicates the uniformity or narrow size distribution, as shown in Figure 2. The zeta potential of the niosome was −15.28 ± 0.16 mV, which indicates that the charged niosomes are stable against aggregation and fusion due to the increasing repulsive forces between the particles in the niosome systems [29]. On multidrug resistance, zeta potential was an important factor in which usually negatively charged particles show good interaction between drugs with the cell membrane during drug penetration [30].

#### 3.3.2. pH Analysis

The pH value obtained for optimum niosome formulation was 6.70 ± 0.01. The pH of the niosome formulation which needs to be nebulized to the lung should be in the range of 6.6 to 6.8 to mimic the condition in the lung cancer area as well as for inhaled drugs [31]. Extremely acidic pH may increase drug loss and result in severe cough and irritation [32].

### 3.4. Drug Entrapment Efficiency

The entrapment efficiency of the niosome is usually affected by the nature of the encapsulated drug and nonionic surfactant used in the preparation of formulation. The entrapment of Gem and Cis was investigated using UV–visible spectroscopy at wavelengths 268 and 207 nm, respectively. The entrapment efficiency values achieved for Gem and Cis were 74.37 ± 0.87% and 85.44 ± 0.55%, respectively. These results showed that the entrapment efficiency of Cis was higher by 11.10% when compared to Gem. This was due to the polarity of the drugs, where Cis is less polar than Gem. Hence, Cis can easily pass through the lipid bilayer membrane. Gem is expected to passively pass through the lipid bilayer membrane and would be trapped in the aqueous core.

Theoretically, niosome formation requires nonionic surfactants as the main component, and the nature of nonionic surfactants such as phase transition temperature and length of alkyl chain also has a huge impact on the entrapment efficiency of the drug. Span 60 has a higher phase transition temperature (53 °C) when compared to Span 20 (−12 °C), which provides better entrapment. When the phase transition temperature is increased from −12.0 to 53.0 °C, it will improve the ability to form a more rigid and less leaky bilayer structure, which will further improve entrapment efficiency [33]. Niosome formulation using Span 60, which has the longest alkyl chain, showed higher entrapment efficiency when compared to the formulations using Span 20 and Span 80 [34].

### 3.5. Morphology

Figure 2b shows spherical-shaped vesicles that exhibited large unilamellar vesicles for optimized niosomes. The particle size measured by TEM ranging between 160.22 and 164.12 nm agreed with the particle size measured by Zetasizer. As shown in the image, a clear spherical layer of the niosome particle was formed. However, imbalanced composition will lead to instability of the niosome, such as aggregations or deformation of shape.

### 3.6. Aerosol Output

The aerosol output obtained from OMRON MicroAIR nebulizer for the optimized niosome formulation was 96.22%, which indicated that the formulation was being successfully delivered by the nebulizer.

The high percentage of the aerosol output can be assigned to increased electrical conductivity, in which the high electrostatic charge present in pure water was suppressed. Another contributing factor is the lower surface tension of the formulation (35.49 ± 0.01 mN/m) compared to pure water (72.16 mN/m). Liquid with low surface tension is less resistant towards aerosolization. Surface tension represents the force that resists the formation of new surface area and a lower surface tension increases the aerosol output [35].

### 3.7. Stability Analysis

Niosome with high stability must reveal constant particle size, no phase separation or precipitation of the membrane component, and have no change in color. The addition of cholesterol to form hydrogen bonds with the hydrophilic head of the surfactant can provide rigidity to the niosome structure, reduce drug leakage, and thus, prolong its stability [36]. The presence of SDS as a stabilizer in the niosome, helps to improve stability by reducing the particle size and increasing the negatively charged zeta potential, causing electrostatic repulsion between niosome particles and preventing aggregation [37]. The centrifugation test can be used to predict shelf life under normal storage conditions by observing the creaming or coalescence of the dispersed phase [23]. After centrifugation at 4000 rpm for 15 min, the optimized NGC formulation showed good physical stability with no phase separation.

**Figure 3:**
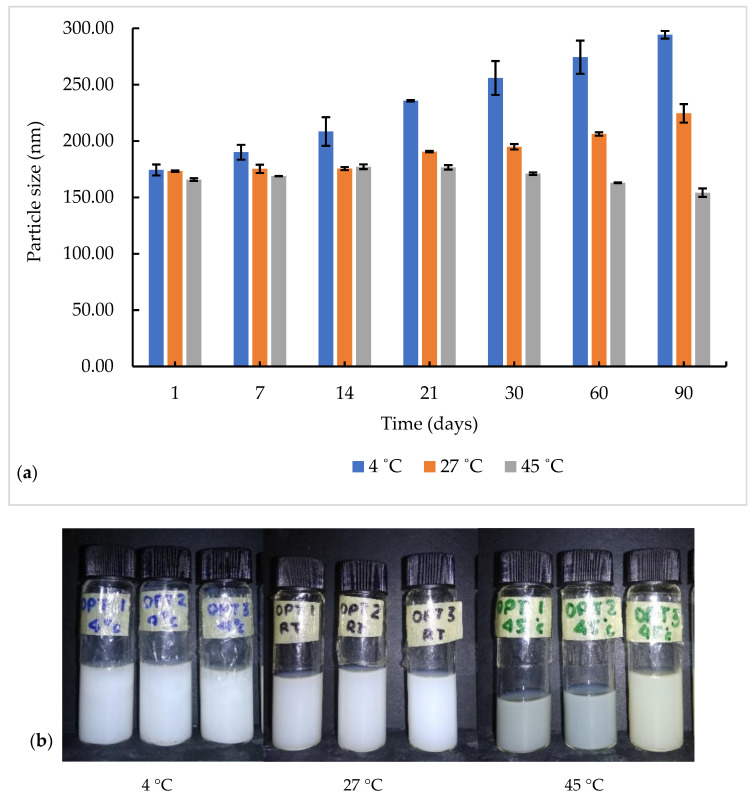
(**a**) Particle size as a function of time at different storage temperatures for 90 days. (**b**) Physical appearance of NGC formulation after 90 days.

A further stability test was performed by storing the optimized NGC formulation at different storage temperatures for 90 days. Interestingly, our niosome formulation was more stable at a high temperature of 45 °C, compared to lower temperatures of 27 and 4 °C (Figure 3a). At 45 °C, the particle size remains within ±15 nm when observed for 90 days, with only a slight change of color from white to light yellow (Figure 3b). The optimized niosome formulation remained physically stable at 27 °C with no precipitation or aggregation observed. Unfortunately, the particle size of the niosome increased gradually for up to 90 days before starting to agglomerate and becoming concentrated when stored at 4 °C. Nagalakshmi et al. [38] reported a different observation, in which the niosome was more stable at low temperature compared high temperature. The heating method (without use of any solvent) as described by Basiri et al. [21], which mixed all the components using a buffer solution that was employed to produce the NGC formulation, may have contributed to this stability. Moreover, the addition of SDS as a stabilizer improves the stability of NGC at 27 °C, which is more convenient for the storage temperature for future use in medical purposes. High temperature could cause drug leakage from the high fluidity of lipid bilayers, which could be seen as the degradation of the color of the optimized niosome formulation at 45 °C [39].

### 3.8. In Vitro Drug Release Analysis

**Figure 4:**
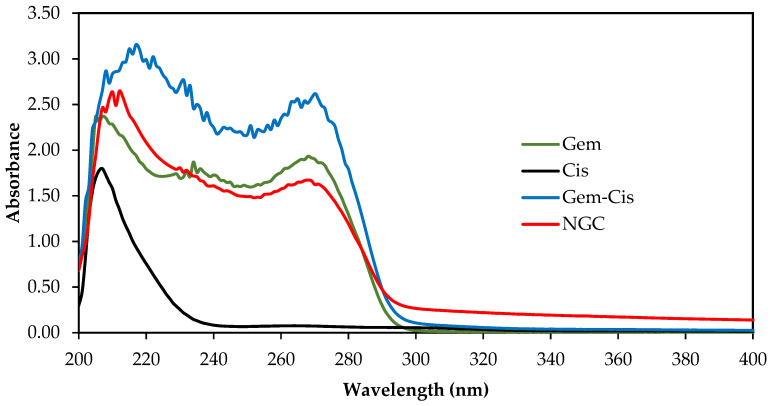
UV–visible spectra of Gem, Cis, Gem–Cis, and the optimized NGC.

The release behavior of Gem and Cis from the optimized NGC was investigated using SLF at different pH (7.4 and 6.7). The UV–visible spectra of Cis, Gem, Cis–Gem, and NGC are shown in Figure 4. The characteristic absorption peaks were found similar to those reported in the literature [40,41]. The calibration curve of absorbance revealed a linear equation with *R*^2^ ~1.000.

**Figure 5:**
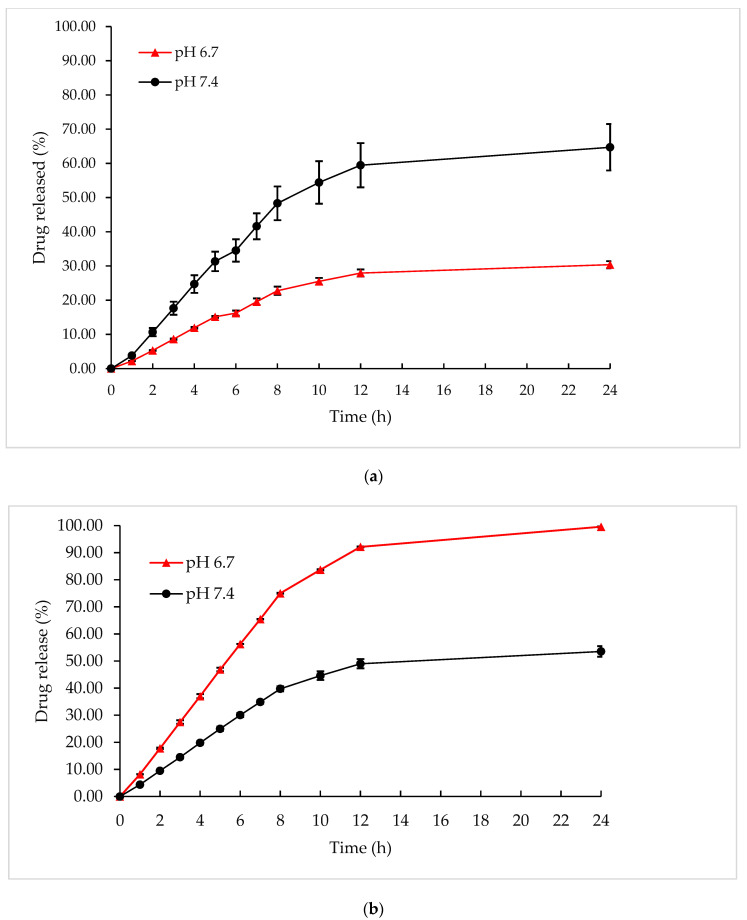
The cumulative release of (**a**) Gem and (**b**) Cis at different pH of SLF.

Figure 5 showed that the drug release was highly pH-dependent and showed a biphasic release with an initial burst release and then, a steady-state of release until 24 h. As shown in Figure 5a, Gem had limited drug release at pH 6.7; 30.38% of the total drugs accumulated in the medium at 24 h. When the pH was increased to pH 7.4, Gem release increased (64.71%). The lower drug release of Gem in the acid condition may be due to Gem having a lower pKa of 3.6 that was unstable and easily degradable in acidic pH [42]. In contrast, it also enhanced the rapid diffusion of Gem release out to the release media at neutral pH [43]. Similar results were obtained by Guo et al. [44], which indicated that at pH 7.4, almost up to 98% of Gem was released when compared to the lower pH.

Figure 5b showed that 99.58% of Cis was released from SLF at pH 6.7 after 24 h of penetration, which was higher compared with Cis released from SLF at pH 7.4 (53.55%). A lower pH enhances the formation of a pore in the niosomal structure due to the solubilization of the lipid bilayer [45]. Thus, Cis from the aqueous core with a small size and no net charge can easily cross the bilayer through dissolution and release at faster rate at acidic conditions [46]. This character of niosome formulation is also useful to reduce the release of Cis from niosomes in normal lung tissue (pH 7.4) and accelerate drug release in acidic conditions in lung cancer tissue (pH 6.6–6.8). As reported by Asmawi et al. [47], drug release was accelerated significantly when under the intracellular tumor environment than the intracellular healthy environment, which was mostly due to the poor water solubility of the drug. The nature of the drug and pH of the release medium are the factors that can affect the percentage of drug release.

The correlation coefficients (*R*^2^) of all kinetic models for Gem and Cis release from the optimized niosome at pH 7.4 and 6.7 are shown in Table 7. The permeation profiles of the niosome formulations followed the Higuchi model for both gemcitabine and cisplatin in SLF pH 7.4 (cumulative percentage of drug release versus square root of time), as the plot showed high linearity, with *R*^2^ values of 0.9114 and 0.9129, respectively. In SLF pH 6.7, the gemcitabine release profile followed the Higuchi model (cumulative percentage of drug release versus square root of time) and the cisplatin release profile followed the Hixson–Crowell model (cube root cumulative amount of drug remaining versus time) with *R*^2^ values of 0.9096 and 0.9974, respectively. The model with *R*^2^ value nearest to one was considered as the best fit release model for the formulation. Drug release that followed the Higuchi model was based on matrix diffusion-controlled release mechanism, while in the Hixson–Crowell model, the release rate is limited by the drug particles’ dissolution rate, which can have higher Cis release in pH 6.7.

### 3.9. In Vitro Cytotoxicity Analysis against MRC5 and A549 Cell Lines

The MTT assay was used to determine the cytotoxicity effect of the optimized NGC against MRC5 and A549 cell lines. The range of the cytotoxic effect was based on the National Cancer Institute and the Geran Protocol: IC_50_ < 21.00 μg/mL (highly toxic), IC_50_ of 21.00–200.00 μg/mL (moderately toxic), IC_50_ of 201.00–500.00 μg/mL (weakly toxic), and IC_50_ values >500.00 μg/mL (non-toxic) [48]. Table 8 showed that the optimized NGC had reduced cytotoxicity effects against both MRC5 and A549 when compared with the control (Gem + Cis alone) from very toxic (IC_50_ < 1.56 µg/mL) to weakly toxic (IC_50_ 280.00 µg/mL) and moderately toxic (IC_50_ = 46.00 µg/mL), respectively, after 72 h of treatment. The blank niosome (formulation without drugs) did not induce cytotoxicity against both cells as the IC_50_ value was not obtained up to the concentration of 500 μg/mL. Thus, the niosome formulation could be used as a potential carrier for chemotherapy treatment, promising fewer side effects.

A combination of Gem and Cis (as a control) clearly appears highly toxic against A549 cells with an IC_50_ value < 1.56 µg/mL, which decreases cell viability from approximately 49.14 to 32.64% of Cis alone. According to Crul et al. [49], Gem and Cis showed good interaction in a combination experiment in lung cancer cells, with the ability to be more potent toward cancer cells, and had the potential to reduce side effects compared to systemic drug release, due to being less toxic in the neutral blood environment [50]. When entering the cell, Cis inhibit tumor growth by directly attacking DNA, which cross-links guanine bases in DNA double-helix. This results in the formation of DNA cross-links preventing the synthesis and replication of DNA, which causes the cell to be in the mode of cell cycle arrest [51]. However, for Gem, it will divide into two active metabolites; gemcitabine diphosphate and gemcitabine triphosphate. The gemcitabine diphosphate inhibits the enzymes, which required for DNA synthesis, while the gemcitabine triphosphate competes with endogenous deoxy nucleoside triphosphates to be incorporated into DNA [49].

## 4. Conclusions

The results of this study demonstrated that the optimized NGC formulation was successfully formulated using a heating method with good stability and homogeneity against phase separation at different temperatures during 90 days of storage. This formulation has appropriate values in controlled drug release, is safe, and exhibits cell growth inhibition against A549 lung cancer cells. These studies suggested that the optimized NGC formulation has the potential to be used for cancer treatment with good entrapment efficiency and aerosol output. However, this should be further confirmed by performing cascade impactor analysis, which reported their benefits to correlate with the deposited drug into the lungs through inhalation.

A synergistic effect between Gem and Cis has been demonstrated in NSCLC, and their response rates are as high as 54%, as reported in the literature. On top of that, the synergistic effect between Gem and Cis in an optimized NGC formulation can be an interesting study to see the changes in their response rates against NSCLC. The unique mechanisms of action of Gem and Cis and their favorable toxicity profile make these drugs interesting prospects for combination chemotherapy.

## Figures and Tables

**Table 1:** Level of independent variable proportions.

Component	Lower Limit (% *w*/*w*)	Higher Limit (% *w*/*w*)
Tween 65: Span 60 (2:1), A	2.00	4.00
Cholesterol, B	0.90	1.20
Glycerol solution, C	60.87	63.17

**Table 2:** Solubility of drug gemcitabine and cisplatin in NaCl solution (0.90% *w*/*v*).

Gem + Cis (Ratio 1:1, % *w*/*w*)	Observation
0.10	Soluble
0.20	Soluble
0.30	Soluble
0.40	Not Soluble

**Table 3:** Predicted and actual values of particle sizes of the composition of the niosome obtained from Design-Expert.

Run	Independent Variables (% *w*/*w*)			Particle Size (nm)	
	T65:S60 (2:1), A	Cholesterol, B	Glycerol Solution, C	Actual Value	Predicted Value
1	2.00	1.20	62.87	238.10	239.43
2	2.00	0.90	63.17	191.60	195.58
3	4.00	1.20	60.87	231.90	230.6
4	3.00	1.05	62.02	189.70	183.13
5	2.50	1.05	62.52	160.20	167.27
6	3.50	0.98	61.60	209.20	207.20
7	2.00	1.05	63.02	166.40	161.00
8	3.00	1.05	62.02	182.20	183.13
9	3.00	1.20	61.87	255.10	253.85
10	2.00	0.90	63.17	198.50	195.58
11	3.00	1.05	62.02	181.70	183.13
12	4.00	0.90	61.17	238.00	280.6
13	3.50	1.13	61.45	244.80	243.90
14	3.00	1.05	62.02	180.40	183.13
15	3.00	0.90	62.17	240.90	240.48
16	4.00	1.05	61.02	241.70	243.67

Note: SDS (0.5% *w*/*w*), NaCl solution (33.33% *w*/*w*), gemcitabine (0.05% *w*/*w*), and cisplatin (0.05% *w*/*w*) were kept constant.

**Table 4:** Analysis of variance (ANOVA) for the model derived by the D-optimal mixture design.

Source	F-Value	*p*-Value
Model	91.6300	<0.0001
Linear Mixture	64.3800	<0.0001
Lack of fit	0.3217	
R-squared (*R*^2^)	0.9874	
Adjusted R-squared (Adj-*R*^2^)	0.9767	
Standard Deviation (SD)	4.9200 ± 0.0070	

**Table 5:** Validation sets of niosome formulation.

Independent Variables (% *w*/*w*)				Particle Size (nm)		RSE (%)
Run	T65:S60 (2:1), A	Cholesterol, B	Glycerol Solution, C	Actual Value	Predicted Value	
1	2.50	0.95	62.62	196.55	193.65	1.50
2	2.50	1.15	62.42	194.20	194.95	1.13
3	3.50	1.05	61.52	211.10	207.02	1.97
4	3.50	1.10	61.47	230.40	228.59	0.79
5	4.00	0.95	61.12	197.90	196.94	0.49
6	2.00	1.13	62.94	186.70	185.29	0.76
7	2.50	1.05	62.52	169.60	168.10	0.89
8	3.00	1.10	61.97	195.80	193.36	1.26

Note: SDS (0.5% *w*/*w*), NaCl solution (33.33% *w*/*w*), gemcitabine (0.05% *w*/*w*), and cisplatin (0.05% *w*/*w*) were kept constant.

**Table 6:** Optimum composition and physicochemical characteristics of the niosome formulation.

**Optimum Niosome Formulation (% *w*/*w*)**	
Tween 65: Span 60 (2:1)	2.00
Cholesterol	1.01
Glycerol solution	63.06
Sodium dodecyl sulphate	0.50
NaCl solution (0.90% *w*/*v* in deionized water)	33.33
Gem	0.05
Cis	0.05
**Physicochemical Characteristics**	
Particles size (nm)	166.45 ± 0.90
Predicted particles size (nm)	159.24
PDI	0.16 ± 0.02
Zeta potential (mV)	−15.28 ± 0.16
pH	6.70 ± 0.01

**Table 7:** The correlation coefficient (*R*^2^) for different models of mechanisms of drug release at different pH of SLF.

Kinetic Model	Correlation Coefficient (*R*^2^)			
	Gem		Cis	
	pH 7.4	pH 6.7	pH 7.4	pH 6.7
Zero-order	0.7469	0.7406	0.7435	0.7463
First-order	0.8163	0.7683	0.8006	0.9882
Higuchi	0.9114	0.9096	0.9129	0.9124
Hixson–Crowell	0.7939	0.7591	0.782	0.9974
Korsmeyer–Peppas	0.8392	0.8775	0.8403	0.8023

**Table 8:** IC_50_ values of the samples after 72 h treatment.

Sample Name	IC_50_ (µg/mL)	
	MRC5	A549
Gem	<1.56	<1.56
Cis	<1.56	66.00
Gem + Cis (Control)	<1.56	<1.56
Blank Niosome (Niosome without drugs)	>500.00	>500.00
Optimized NGC (Niosome containing Gem and Cis)	280.00	46.00
